# Stereochemistry of the Reaction Intermediates of Prolinol Ether Catalyzed Reactions Characterized by Vibrational Circular Dichroism Spectroscopy

**DOI:** 10.1002/chem.201905614

**Published:** 2020-02-04

**Authors:** Tino P. Golub, Christian Merten

**Affiliations:** ^1^ Fakultät für Chemie und Biochemie, Organische Chemie II Ruhr Universität Bochum Universitätsstrasse 150 44801 Bochum Germany

**Keywords:** asymmetric catalysis, chirality, circular dichroism, enamines, vibrational spectroscopy

## Abstract

Spectroscopic characterizations of key reaction intermediates are often considered the final confirmation of a reaction mechanism. This proof‐of‐principle study showcases the application of vibrational circular dichroism (VCD) spectroscopy for the characterization of in situ generated reaction intermediates using the key intermediates of enamine catalysis of Jørgensen–Hayashi‐type prolinol ether catalysts as model system. By comparison of experimental and computed spectra, the enamines are shown to preferentially adopt an anti‐conformation with *E*‐configured C=C bond. For the parent prolinol catalyst, the structure and stereochemistry of the oxazolidine side product is determined as well. This study thus demonstrates that VCD spectra can provide insights into structural preferences of organocatalysts that utilize a covalent activation mechanism. Thereby it outlines new fields of applications for VCD spectroscopy and finally adds the technique to the toolbox of physical organic chemistry for in‐depth mechanistic studies.

## Graphical abstract

**Before it reacts**: The enamine and oxazolidine intermediates are characterized by in situ IR and VCD spectroscopy. The study demonstrates the proof‐of‐principle for such analysis of asymmetric catalysts employing a covalent substrate activation mechanism by VCD spectroscopy.Stereochemistry of the reaction intermediates of prolinol ether catalyzed reactions characterized by vibrational circular dichroism spectroscopy (Merten and Golub @MertenLab @ruhrunibochum)

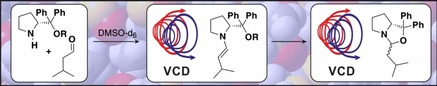

In the rapidly growing field of asymmetric catalysis, detailed knowledge about the relative orientation of reactants and catalysts is very important as the success of the stereo‐induction step crucially depends on the exact positioning of the reactant in the chiral field of the catalyst. Therefore, any insights into the structure of reactive intermediates or active conformations of catalysts foster an understanding of the underlying reaction mechanisms and, in the long term, lead to an improvement of the catalyst design. Besides computational methods,^1^ solution‐phase spectroscopic techniques such as NMR spectroscopy are typically used to gain information about the formation and stability of intermediate species such as hydrogen bonded complexes and covalently activated substrates. However, there is often no direct link between the computational results and NMR spectroscopic data. In fact, conformational preferences are mostly derived from comprehensive scalar and through‐space coupling analysis using methods such as COSY and NOESY before they are qualitatively compared to computationally predicted structures.

Vibrational circular dichroism (VCD) spectroscopy is the chiroptical version of IR spectroscopy and measures the differential absorbance of left‐ and right‐circularly polarized light during vibrational transitions.^2^ As it does not rely on specific UV/Vis chromophores, it has become a versatile and powerful tool for the determination of absolute configurations.^3^ Moreover, as vibrational spectroscopic technique, VCD spectroscopy has shown a remarkable sensitivity for solute–solvent and solute–solute interactions.^4^ All these applications rely on the direct comparison of experimental and computed spectra and a good match directly confirms the predicted conformational preferences.^3^ In several studies, we could already show that VCD spectroscopy can significantly contribute to a better understanding of stereochemical information transfer in asymmetric catalysis. Our initial study in this field focused on an ion‐pair based catalyst system which consisted of an achiral salen‐manganese cation and a chiral BINOL‐derived phosphate.^5^ The spectral analysis showed that the phosphate binds to the cation and thereby induces a preference for a chiral conformation to it. The intensities of VCD spectral signatures associated with vibrations of the cation were found to correlate with stereo‐selectivities observed in epoxidation reactions. Subsequently, we focused on hydrogen‐bonding organocatalysis with chiral thiourea catalysts. For a model thiourea^6^ and the bifunctional thiourea catalyst developed by Takemoto,^7^ we revealed unexpected binding motifs^6^ and interesting challenges in their computational prediction.7a, 8

After having demonstrated the applicability of VCD spectroscopy for ionic and hydrogen bonding organocatalysts, we expected that valuable information about active conformations could also be derived for catalysts that utilize a covalent substrate activation mechanism. This mode of activation, however, is particularly challenging to study by VCD spectroscopy as the technique generally requires relatively high concentrations, while the concentration of a covalently activated substrate, that is, the substrate‐catalyst complex of interest, is often quite low due to subsequent (intramolecular) reactions. Hence, a proof‐of‐principle study required a model system in which the active substrate‐catalyst complex can be generated in high yields and which does not react (too fast) during the course of the measurement.

The search for a suitable model system has drawn our attention to enamine catalysis^9^, ^10^ based on prolinol and prolinol ethers and in particular to the model reaction shown in Scheme 1. Following the generally accepted reaction mechanism of enamine catalysis proposed by Houk and List,10d the prolinol ethers **1 a/b** and prolinol **1 c** itself react with isovaleraldehyde to enamine **2**, the active structure of the catalytic cycle.^9^ This transformation activates the former α‐position of the aldehyde for addition reactions to nucleophiles as second reactants. According to extensive NMR spectroscopic data on the kinetics of the formation of these enamines reported by Gschwind and co‐workers,^9^, 10b, 10c, 11 **2 a**–**c** are formed immediately after mixing of equimolar amounts of **1 a**–**c** and the aldehyde in [D_6_]DMSO. Yet, the enamines feature different stabilities: Enamine **2 a** is obtained in about 85 % yield and stable for a long period of time (more than 8 hours). The TMS‐derivative **2 b** is formed in about 75 % yield, but tends to react further towards oxazolidine **3** over several hours, thus decreasing the concentration of **2 b**. The prolinol enamine **2 c** is not obtained in significant yields as the reaction towards **3** takes place almost immediately. The described transformation of the enamine species **2** to oxazolidine **3**, which passed through an iminium intermediate, is considered an undesired side reaction of the catalytic cycle as it withdraws enamine from the reaction equilibrium. Consequently, under catalytic conditions, its formation leads to a decrease in catalyst turnover as **3** does not participate in the catalytic cycle anymore.^12^

**Scheme 1:**
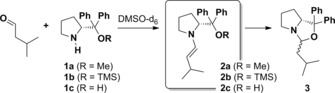
Reaction of isovaleraldehyde with prolinol‐based catalysts **1 a**–**c** gives the enamines **2 a**–**c**. In an undesired side reaction, which occurs mainly in absence of nucleophilic reagents, the enamine may react further to **3** via an iminium intermediate (not shown). This side reaction proceeds rapidly for **2 c**, slowly for **2 b** and it is not detected for **2 a**.^9^

The kinetic profiles of enamine formation of the three catalysts **1 a**–**c** made them ideal model systems for this proof‐of‐principle VCD study on covalent substrate activation. In fact, depending on the choice of the catalyst, we get VCD spectroscopic access to two key intermediates of the catalytic cycle, that is, the enamine and the oxazolidine side product. This is demonstrated in Figure 1, in which each panel shows the IR and VCD spectra of a catalyst (**1 a**–**c**) and the respective reaction intermediates derived therefrom by preparing equimolar mixtures of the catalyst with isovaleraldehyde. As the enamines **2 a** and **2 b** are formed from **1 a/b** almost instantaneously and in high yields, the VCD spectra of **2 a/b** were recorded after a short equilibration period of 15 minutes after mixing. The VCD spectra of **2 a** were recorded over the course of 4 hours using the same sample. Due to the slow rearrangement of **2 b** to **3**, the VCD spectra of **2 b** were obtained by averaging over four 1 hour measurements of freshly prepared samples. In order to follow the rearrangement reaction, one of the samples of **2 b** was left on the bench for 72 hours in the cuvette, which subsequently allowed us to record also the spectrum of the resulting rearrangement product **3**. As the enamine **2 c** undergoes an immediate rearrangement reaction to **3**, the mixture of **1 c** with isovaleraldehyde gave the spectra of pure oxazolidine.

**Figure 1:**
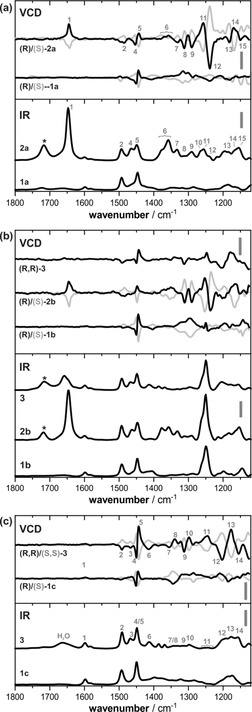
Comparison of the experimental spectra of catalysts **1 a**–**c** and the reaction intermediates **2 b/c** and **3**. All spectra were recorded in [D_6_]DMSO at concentrations of 0.3–0.4 m and 100 μm pathlength. The scaling bars represent intensities of 10^2^ m ^−1^ cm^−1^ in the IR and 10^−2^ m ^−1^ cm^−1^ in the VCD spectra. The asterisk marks the carbonyl C=O stretching vibration of residual aldehyde. Numbers denote band assignments discussed in the text and in Figures 3 and 5.

The overview in Figure 1 nicely highlights the spectral differences between pure catalysts and the reaction intermediates. Without going into detail on band assignments, a few spectral signatures can easily be traced to certain species. The band at 1650 cm^−1^ in the IR spectra (band 1) of the enamines can be assigned to the C=C stretching vibration of the enamine, for instance. Similarly, all IR spectra in Figure 1 b feature a strong band at 1250 cm^−1^, which arises from the C−Si stretching vibration of the TMS group in **1 b**/**2 b**. Further empirical band assignments can be made: The IR spectra of the enamines **2 a/b** and the oxazolidine **3** derived from **2 b** also show a small band at 1720 cm^−1^ which can be assigned to residual aldehyde still present in the sample solution. In the reaction **1 c**→[**2 c**]→**3**, all aldehyde is consumed during the rearrangement reaction, so that the aldehyde band is not observed. Instead, the weak broad band at 1660 cm^−1^ of the water bending mode can be observed which arises from the about two equivalents of water produced during the formation of the enamine and the ring closure reaction.

It has to be noted that the VCD spectral signatures are even more characteristic for the different species than the IR signatures. In fact, significant similarities between the VCD spectra of the two investigated enamines can be identified (a direct comparison of the two sets of spectra is provided in Figure S1 of the Supporting Information). Besides the positive band of the C=C stretching mode (band 1), these are the characteristic (−/+/−) pattern in the range of 1340–1290 cm^−1^ (bands 8/9) and the bisignate (+/−) VCD signature centered at 1250 cm^−1^ (bands 11/12). The TMS‐group with its C−Si stretching vibration at 1250 cm^−1^, which is dominant in the IR spectra of the series **1 b**→**2 b**→**3**, does not seem to have significant influence on the overall VCD spectral pattern of the enamine species.

The experimental spectra in Figure 1 show that the intermediates can easily be distinguished experimentally. From a mechanistic perspective, however, it is not only important to distinguish the different species but also to characterize their conformational preferences. In particular, it is important to establish the preferred spatial orientation of the C=C bond, that is, if it points towards (*syn*) or away from (anti) the diphenyl ether side chains, as well as its configuration (*E*/*Z*). While the latter is easily derived from ^3^ *J*‐coupling constant analysis in ^1^H NMR spectra and for **2 a/b** known to be *E*,^9^ information about the *syn*/*anti* orientation can only be derived from {^1^H,^1^H}‐NOESY and {^1^H,^13^C}‐HMBC experiments.9b As we show in the following, direct access to this information can be obtained by comparing the experimental VCD spectra with computed spectra.

A comprehensive conformational analysis is the basis for reliable spectra simulations. The conformational search for enamine **2 a** required the systematic evaluation of nine dihedral angles and rotatable bonds that define the conformational space of the molecule. Briefly, these are the torsional angles determining the conformations and relative spatial orientations of the alkyl and the diphenyl ether side chains and two torsional angles defining the ring puckering of the pyrrolidine ring. At the B3LYP/6‐31G+(2d,p)/IEFPCM(DMSO) level of theory, the search led to a total of 105 unique conformers of which three are almost identical in energy (full list including geometries can be found in the Supporting Information). Figure 2 shows two of them denoted **2 a** *‐c1* and *‐c2*, which differ by the spatial orientation of the C(Ph)_2_OMe side group (i.e. the torsional angle NC*CO). The third highly populated conformer differs from **2 a** *‐c1* only in the spatial orientation of the methoxy group (torsional angle spanning C*‐C(Ph)_2_‐*O*‐CH_3_ is −76° instead of ≈180°). Together these three conformers contribute equally to total of about 70 % to the Boltzmann population. It should be noted that, in agreement with previous NMR studies,^9^ the *syn*‐conformers and the *Z*‐isomers are predicted to have negligible contributions to the conformational equilibrium (0.3 % respectively 0.1 % according to Δ*E*_ZPC_).

**Figure 2:**
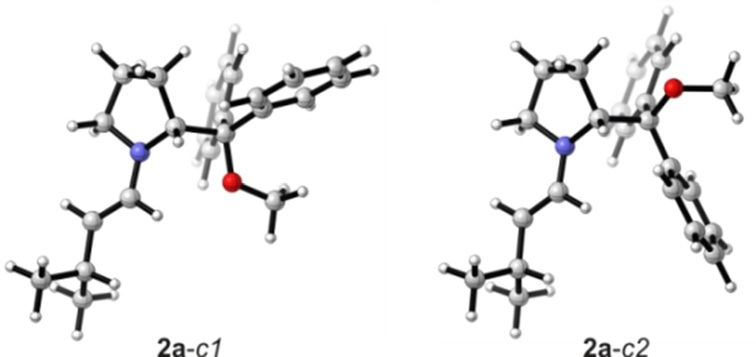
The computed lowest energy conformers *c1* and *c2* of **2 a**, which only differ in the torsional angle NC*CO (−62° and ≈180°).

Based on the full set of conformers, population averaged IR and VCD spectra of **2 a** were calculated by co‐adding the respective single‐conformer spectra multiplied by their corresponding Boltzmann weights (according to Δ*E*_ZPC_). Thereby, the contribution of each conformer is weighted and the spectral intensities are scaled accordingly. Following this procedure, the calculated spectra shown in Figure 3 were obtained. Already on first sight, an almost perfect match between experiment and theory can be noted. All experimental VCD spectral features are nicely reproduced. Solely in the IR spectra, there are some bands that appear to be overestimated in intensity which may, however, simply be related to the uniform line width used to simulate the spectra.

**Figure 3:**
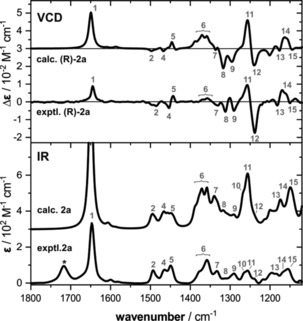
Comparison of the experimental and computed IR and VCD spectra of **2 a**. The measurement was carried out for a solution of an equimolar mixture of **1 a** and isovaleraldehyde in [D_6_]DMSO (0.37 m, 100 μm path length) after 15 minutes of equilibration. Spectra were computed at the B3LYP/6‐31+G(2d,p)/IEFPCM(DMSO) level of theory. Numbers indicate band assignments, the asterisk marks the residual band of unreacted aldehyde.

The good match between the experimental and theoretical spectra confirms the validity of the computed conformational distribution. In fact, considering only the *Z*‐ or the *syn*‐conformers in the prediction of the VCD signatures, that is, making them the major species in the conformational distribution, yields VCD spectra that are clearly different from the experimentally observed ones (cf. Supporting Information Figure S2). Consequently, the lowest energy conformers shown in Figure 2 can be assumed to be the major species in solution without the need to analyse and interpret NOE contacts and simply by directly comparing experimental and computed spectra.

The computed spectra can also be used to assign bands in the IR and VCD spectra to specific vibrational motions. This is of particular interest for the spectral signatures of bands 8–12 that have been identified as characteristic for the enamines. Although the vibrational motions in this range appear complex upon visual inspection, bands 8 and 9 can qualitatively be assigned to CH_2_‐wagging and =C−H in‐plane bending motions. Bands 10 and 11 arise from in‐phase and out‐of‐phase C_tert_−H deformation modes at the stereocenter and the isopropyl group. Interestingly, band 10 does not contribute much to the VCD spectral signature although it is quite strong in the IR. In contrast, the weak band 12, which is best described as CH_2_‐twisting motions localized in the five‐membered ring, shows a very strong VCD band. As the computational analysis of the IR and VCD spectra of **2 b** leads to essentially the same quality of match and the same conclusions (cf. Figure S3), these band assignments can be considered general for the enamines.

As mentioned above, the oxazolidine **3** is produced in an unwanted side reaction for equimolar mixtures of **1 a** and isovaleraldehyde almost quantitatively. After 72 hours it was observed as main species in solutions of **1 b** with the aldehyde. Previous NMR‐based reports on the stereochemistry of **3** 9a, 13 can be confirmed by carrying out a systematic conformational analysis for both diastereomers of the oxazolidine, (*R*,*R*)‐ and (*R*,*S*)‐**3**. In contrast to **2 a**, the conformational space of **3** is significantly smaller as only the alkyl chain conformations had to be considered. Hence, we found seven conformers for each of the stereoisomers; the lowest energy conformer of (*R*,*R*)‐**3** is shown in Figure 4 and the energetic and structural data for both isomers is summarized in the Supporting Information.

**Figure 4:**
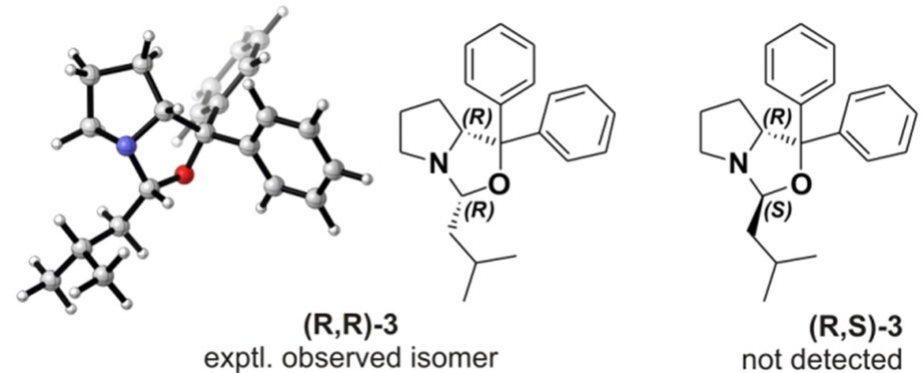
The structures of the (*R*,*R*)‐ and (*R*,*S*)‐**3** diastereomers. Only the (*R*,*R*)‐isomer is observed experimentally by VCD spectroscopy.

The comparison of the experimental and computed VCD and IR spectra in Figure 5 presents a very good match between the experimental spectra and those predicted for (*R*,*R*)‐**3**. In particular the VCD spectral pattern is very nicely reproduced by the calculations. Even in the range from 1350–1250 cm^−1^ (bands 7–11)_,_ where the IR spectrum features a broad and little defined band pattern, the sharp VCD signatures match very well. This range is also suitable to distinguish the (*R*,*R*)‐ and the (*R*,*S*)‐stereoisomers (Figure S4): For (*R*,*S*)‐**3**, bands 9 and 10 are inverted in sign so that a clear differentiation of the stereoisomers is possible.

**Figure 5:**
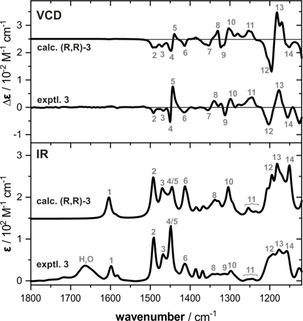
Comparison of the experimental IR and VCD spectra of **3** derived from **1 c** and isovaleraldehyde with those computed for (*R*,*R*)‐**3**. The measurement was carried out for a solution of an equimolar mixture of **1 c** and the aldehyde in [D_6_]DMSO (0.31 m, 100 μm path length) after 15 minutes of equilibration. Spectra were computed at the B3LYP/6‐31+G(2d,p)/IEFPCM(DMSO) level of theory. Numbers indicate band assignments.

In summary, this study describes the VCD spectroscopic characterization of reaction intermediates of the prolinol ether catalytic cycle. As such it is the first VCD study on an asymmetric catalyst that operates by covalently activating the substrate, which presents a particular challenging target as such species typically have limited lifetimes. Given the prior knowledge on the kinetics of the reaction,9a the timeframes in which the intermediates could be captured were known and we could show that all important structural information about the intermediates such as *E*/*Z* and *syn*/*anti* conformational preferences could be directly confirmed by comparison of the experimental spectra with computed ones. Thereby we could show that VCD spectroscopy has the potential to become a powerful complementary technique to NMR spectroscopy for the characterization of reaction intermediates. Hence, this combined approach is highly promising and should be applicable to all systems for which the target species reaches a reasonably high concentration and lifetime in a steady state. In order to be able to capture the VCD signatures of unknown short(er)‐lived species, further instrumental developments are required that allow for higher time‐resolution^14^ respectively significantly faster measurements.^15^

## Conflict of interest

The authors declare no conflict of interest.

## Supporting information

As a service to our authors and readers, this journal provides supporting information supplied by the authors. Such materials are peer reviewed and may be re‐organized for online delivery, but are not copy‐edited or typeset. Technical support issues arising from supporting information (other than missing files) should be addressed to the authors.

Supplementary

## References

[1a] T. Sperger , I. A. Sanhueza , F. Schoenebeck , Acc. Chem. Res. 2016, 49, 1311–1319; 27171796 10.1021/acs.accounts.6b00068

[1b] P. H.-Y. Cheong , C. Y. Legault , J. M. Um , N. Çelebi-Ölçüm , K. N. Houk , Chem. Rev. 2011, 111, 5042–5137. 21707120 10.1021/cr100212h PMC3154597

[2] L. A. Nafie , Vibrational Optical Actvity, Wiley, UK, 2011.

[3] C. Merten , T. P. Golub , N. M. Kreienborg , J. Org. Chem. 2019, 84, 8797–8814. 31046276 10.1021/acs.joc.9b00466

[4] C. Merten , Phys. Chem. Chem. Phys. 2017, 19, 18803–18812. 28561838 10.1039/c7cp02544k

[5] C. Merten , C. H. Pollok , S. Liao , B. List , Angew. Chem. Int. Ed. 2015, 54, 8841–8845; 10.1002/anie.201501271 26101152

[6] N. M. Kreienborg , C. H. Pollok , C. Merten , Chem. Eur. J. 2016, 22, 12455–12463. 27457499 10.1002/chem.201602097

[7a] N. M. Kreienborg , C. Merten , Chem. Eur. J. 2018, 24, 17948–17954; 30230065 10.1002/chem.201804230

[7b] T. Okino , Y. Hoashi , Y. Takemoto , J. Am. Chem. Soc. 2003, 125, 12672–12673. 14558791 10.1021/ja036972z

[8] N. M. Kreienborg , C. Merten , Phys. Chem. Chem. Phys. 2019, 21, 3506–3511. 29863202 10.1039/c8cp02395f

[9a] M. B. Schmid , K. Zeitler , R. M. Gschwind , J. Am. Chem. Soc. 2011, 133, 7065–7074; 21500780 10.1021/ja111544b

[9b] M. B. Schmid , K. Zeitler , R. M. Gschwind , Chem. Sci. 2011, 2, 1793–1803.

[10a] S. Mukherjee , J. W. Yang , S. Hoffmann , B. List , Chem. Rev. 2007, 107, 5471–5569; 18072803 10.1021/cr0684016

[10b] M. H. Haindl , J. Hioe , R. M. Gschwind , J. Am. Chem. Soc. 2015, 137, 12835–12842; 26388105 10.1021/jacs.5b03420

[10c] M. B. Schmid , K. Zeitler , R. M. Gschwind , J. Org. Chem. 2011, 76, 3005–3015; 21446689 10.1021/jo200431v

[10d] S. Bahmanyar , K. N. Houk , H. J. Martin , B. List , J. Am. Chem. Soc. 2003, 125, 2475–2479; 12603135 10.1021/ja028812d

[10e] U. Grošelj , D. Seebach , D. M. Badine , W. B. Schweizer , A. K. Beck , I. Krossing , P. Klose , Y. Hayashi , T. Uchimaru , Helv. Chim. Acta 2009, 92, 1225–1259.

[11] A. Seegerer , J. Hioe , M. M. Hammer , F. Morana , P. J. W. Fuchs , R. M. Gschwind , J. Am. Chem. Soc. 2016, 138, 9864–9873. 27430865 10.1021/jacs.6b04008

[12] J. Franzén , M. Marigo , D. Fielenbach , T. C. Wabnitz , A. Kjærsgaard , K. A. Jørgensen , J. Am. Chem. Soc. 2005, 127, 18296–18304. 16366584 10.1021/ja056120u

[13a] G. Zuo , Q. Zhang , J. Xu , Heteroat. Chem. 2003, 14, 42–45;

[13b] Y. Okuyama , H. Nakano , H. Hongo , Tetrahedron: Asymmetry 2000, 11, 1193–1198.

[14] J. Helbing , M. Bonmarin , J. Chem. Phys. 2009, 131, 174507. 19895025 10.1063/1.3256224

[15] A. Rüther , M. Pfeifer , V. A. Lórenz-Fonfría , S. Lüdeke , Chirality 2014, 26, 490–496. 24623312 10.1002/chir.22307

