# Supplementary material for: Stereochemistry of the Reaction Intermediates of Prolinol Ether Catalyzed Reactions Characterized by Vibrational Circular Dichroism Spectroscopy
Source: Chemistry. 2020 Feb 4;26(11):2349–53. doi: 10.1002/chem.201905614 (PMC7065232; doi:10.1002/chem.201905614)
Supplement: Supplementary file 1 — Supplementary [file CHEM-26-2349-s001.pdf]

CHEMISTRY
A European Journal

Supporting Information

Stereochemistry of the Reaction Intermediates of Prolinol Ether Catalyzed Reactions Characterized by Vibrational Circular Dichroism Spectroscopy

Tino P. Golub and Christian Merten*[a]

chem_201905614_sm_miscellaneous_information.pdf

Supporting information for

Stereochemistry of the reaction intermediates of prolinol ether catalyzed reactions characterized by VCD spectroscopy

Tino P. Golub,[a] Christian Merten*[a]

Ruhr Universität Bochum,
Lehrstuhl für Organische Chemie II
Universitätsstraße 150
44801 Bochum, Germany
christian.merten@ruhr-uni-bochum.de

Contents

1. Experimental details 2
2. Additional spectra and figures 3
3. Conformational analysis 5
4. Selected Cartesian coordinates 11
5. Additional references 12

1. Experimental details

Materials and sample preparation. Enantiopure samples of both enantiomers of **1a-c** were obtained from Sigma Aldrich (Germany) and used without further purification. Isovaleraldehyde has been obtained from Sigma Aldrich (Germany) and freshly been distilled before use. DMSO-d_6 was obtained from Eurisotop (France). Equimolar mixtures of the prolinol derivatives **1a-c** and the aldehyde were freshly prepared before every measurement.

IR and VCD spectroscopy. The IR and VCD spectra were recorded on a Bruker Vertex 70/ PMA 50 VCD spectrometer. Samples were held in a transmission cell with BaF_2 windows with 100 µm path length. Sample concentrations were adjusted so that the IR absorbance spectrum is below 0.9 absorbance units. Both IR and VCD spectra were generally recorded at 4 cm^{-1} spectral resolution by accumulating 32 respectively at least ~16000 scans (minimum of 4 hours accumulation time for VCD). Baseline correction of the VCD spectra was done by subtraction of the spectra of the corresponding racemic mixtures or the solvent recorded under identical conditions. Throughout the main text, it is referred to the experimental spectra of the *(R)*-enantiomers for comparison purposes.

Computational details. Geometry optimizations and frequency calculations were performed at the B3LYP/6-31G+(2d,p) level of theory using the Gaussian 09 E.01 software package with tight convergence criteria and ultrafine integration grids.[1] Solvent effects were taken into account implicitly by using the integral equation formalism of the polarizable continuum model (IEFPCM)[2] for DMSO. Vibrational line broadening was simulated by assigning a Lorentzian band shape with half-width at half-height of 6 cm^{-1} to the calculated dipole and rotational strength. The calculated frequencies were scaled by a factor of σ=0.98. The theoretical spectra shown in the main text are simulated according to the Boltzmann weights for the relative zero-point corrected energies ΔE_{ZPC} as they were found to be either virtually identical or to agree better with the experimental data than spectra predicted based on Gibbs free energies ΔG_{298K}. Figures are prepared using CylView.[3]

2. Additional spectra and figures

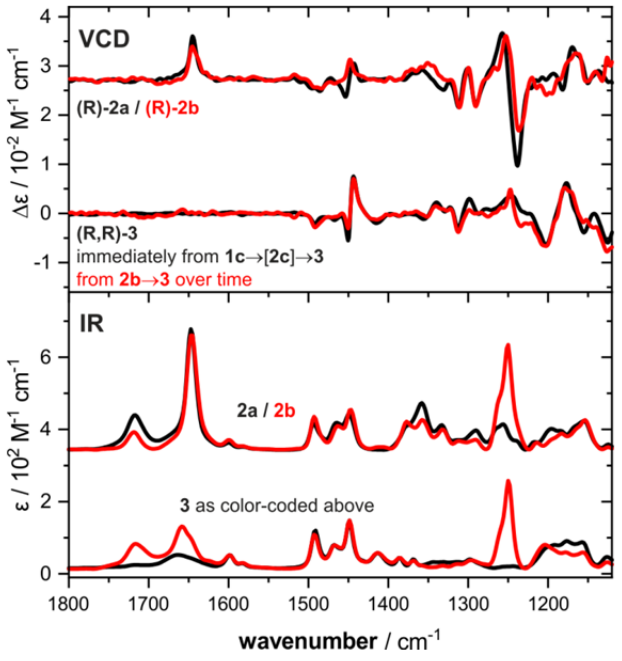

Figure S1. Comparison of the experimental IR and VCD spectra of the enamines **2a** and **2b** and of the oxazolidine obtained directly from **1c** and over time from **2b**.

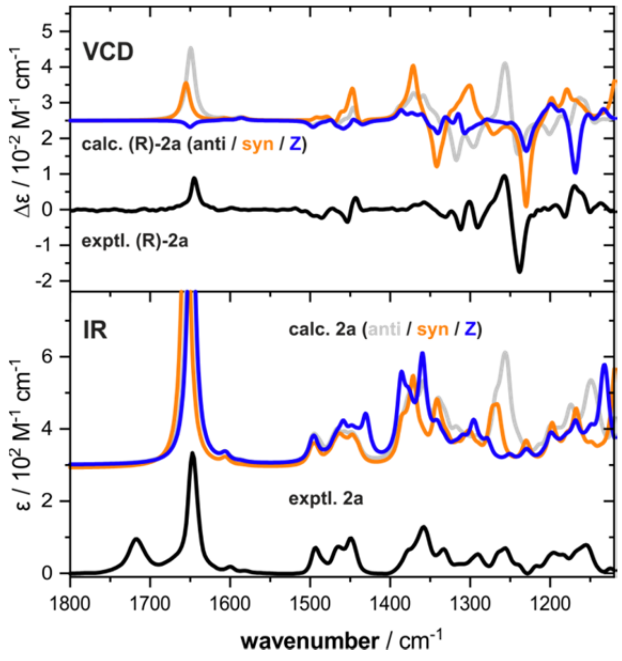

Figure S2. Comparison of the experimental spectra of enamine **2a** with those computed for conformers featuring only the anti-, the syn- or the Z-conformation.

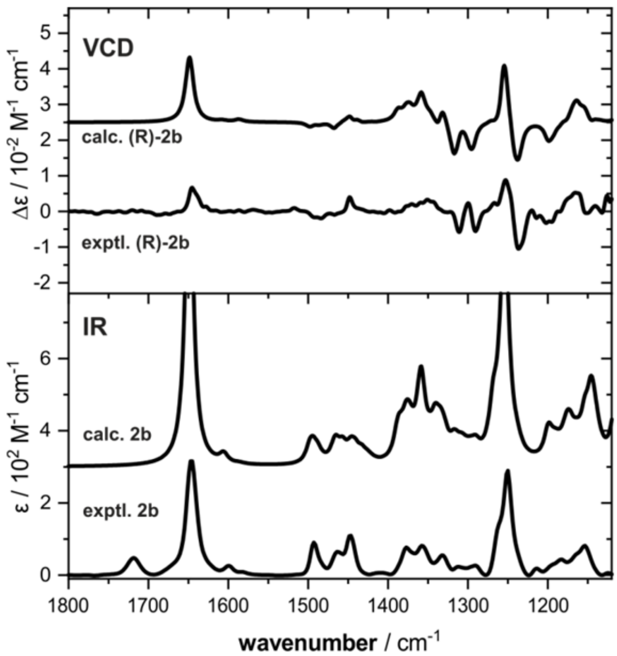

Figure S3. Comparison of the experimental and computed IR and VCD spectra of enamine **2b**.

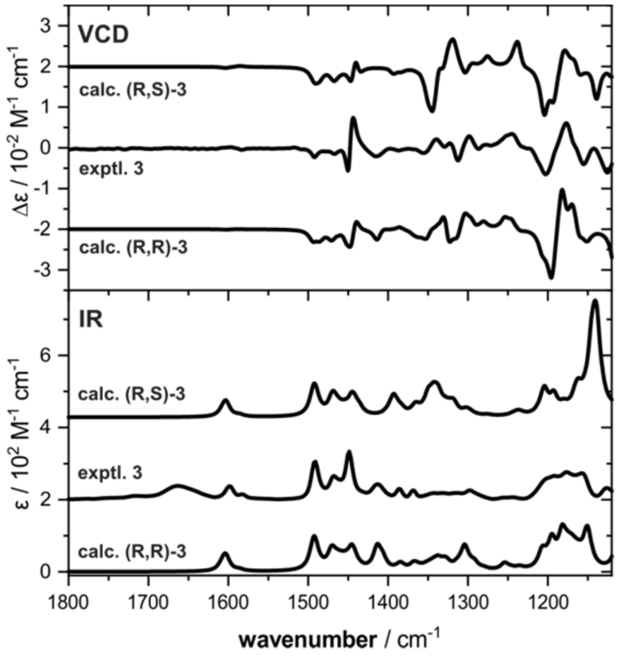

Figure S4. Comparison of the experimental IR and VCD spectra of **3** with those computed for the (R,R)- and (R,S)-**3** diastereomers.

3. Conformational analysis

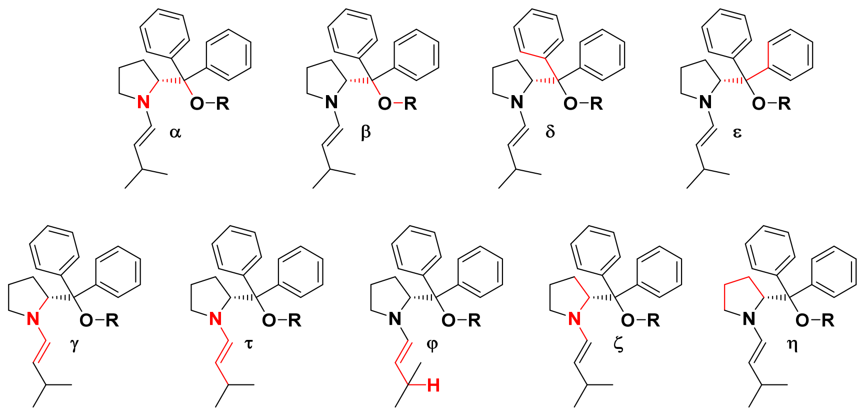

Scheme S1. Angle definition for the enamines **2**.

Table S1. Key torsional angles of the considered conformers of (R)-**1b** and the corresponding relative zero-point corrected and Gibbs Free energies, ΔE_{ZPC} and ΔG_{298K}, and resulting Boltzmann populations (popΔE and popΔG). Torsional angles are given in degrees, relative energies in kcal/mol and populations in percentage. The angles are defined in Scheme S1.

Conf.	A	β	δ	ε	γ	τ	ϕ	ζ	η	ΔE_{ZPC}	ΔG_{298K}	popΔE	popΔG
2a-c1	-61.6	-175.0	90.3	24.1	163.2	178.8	1.3	-147.4	31.1	0.000	0.0	24.4	27.6
2a-c2	177.2	165.2	133.0	-71.6	165.8	179.2	0.8	-148.1	31.2	0.004	0.2	24.2	20.0
2a-c3	-54.6	-76.1	92.8	6.0	163.8	178.7	1.7	-148.3	31.4	0.014	0.1	23.8	21.9
2a-c4	-67.0	-173.5	87.9	31.6	177.1	-178.0	-1.7	-154.3	-30.5	1.0	0.8	4.4	7.5
2a-c5	172.8	164.6	134.1	-70.9	179.0	-178.1	-2.0	-161.2	-32.9	1.0	1.0	4.2	4.7
2a-c6	-61.6	-175.2	90.6	23.9	164.1	179.2	-118.2	-147.7	30.9	1.3	1.4	2.8	2.3
2a-c7	-54.9	-76.1	92.8	6.1	164.2	179.1	-118.0	-148.3	31.3	1.3	1.6	2.6	1.9
2a-c8	-61.8	-175.1	90.3	24.1	163.0	178.5	121.4	-147.4	31.1	1.3	1.5	2.5	2.0
2a-c9	177.0	165.2	133.6	-71.7	165.4	178.9	121.4	-147.7	31.3	1.4	1.8	2.4	1.4
2a-c10	-54.8	-76.2	92.8	6.1	163.4	178.5	121.8	-148.1	31.4	1.4	1.7	2.3	1.5
2a-c11	-58.7	-75.7	92.0	11.0	179.9	-177.4	-1.6	-155.4	-28.0	1.6	1.4	1.6	2.7
2a-c12	167.5	60.0	-30.2	-73.4	165.3	178.8	0.8	-148.0	31.5	1.9	1.8	0.9	1.2
2a-c13	-56.5	174.6	144.0	-84.0	175.5	-178.7	-1.2	-161.4	-34.7	2.1	1.9	0.6	1.1
2a-c14	-67.5	-173.4	87.7	31.9	177.9	-177.7	-120.1	-155.1	-30.5	2.2	2.1	0.5	0.7
2a-c15	172.4	164.8	134.4	-71.1	-179.9	-177.4	-120.1	-161.0	-32.9	2.3	2.5	0.5	0.4
2a-c16	-67.2	-173.6	88.0	31.6	177.4	-178.3	118.8	-154.9	-30.4	2.3	2.2	0.5	0.7
2a-c17	172.4	164.8	134.7	-71.1	179.3	-178.2	119.1	-161.1	-32.9	2.4	2.7	0.4	0.3
2a-c18	163.9	66.6	-27.5	-74.4	178.5	-178.3	-1.8	-161.0	-32.9	2.7	2.4	0.2	0.4
2a-c19	-67.6	-172.9	-88.7	-154.1	-23.8	-180.0	-0.3	-129.2	-29.1	2.7	2.6	0.2	0.3
2a-c20	55.8	-68.1	62.0	-30.9	178.8	-178.7	-1.2	-171.2	-35.1	2.8	2.6	0.2	0.3
2a-c21	-59.1	-75.6	92.0	11.4	-179.1	-177.0	-120.4	-156.0	-27.9	2.9	2.8	0.2	0.2
2a-c22	-58.9	-75.6	91.9	11.1	-179.9	-177.6	118.6	-155.5	-27.9	3.0	3.0	0.1	0.2
2a-c23	167.3	60.2	-30.2	-73.5	165.9	179.2	-118.3	-148.1	31.6	3.2	3.3	0.1	0.1
2a-c24	167.1	60.1	-29.8	-73.6	164.7	178.5	121.1	-147.8	31.6	3.3	3.3	0.1	0.1
2a-c25	-57.2	174.6	145.0	-84.6	176.1	-178.4	-120.1	-162.5	-34.8	3.3	3.1	0.1	0.1
2a-c26	-56.9	174.8	145.1	-84.8	175.4	-179.0	119.4	-162.2	-34.7	3.4	3.1	0.1	0.1
2a-c27	163.7	66.5	-27.6	-74.5	179.8	-177.8	-120.3	-161.2	-32.8	4.0	3.9	0.0	0.0

2a-c28	64.5	-167.0	62.3	-118.6	-24.7	-179.7	0.4	-122.7	-28.1	4.0	4.2	0.0	0.0
2a-c29	-67.1	-173.1	87.3	32.1	-23.5	180.0	119.4	-129.3	-29.0	4.0	4.2	0.0	0.0
2a-c30	163.7	66.7	-27.7	-74.3	178.9	-178.4	118.9	-161.3	-33.0	4.1	4.0	0.0	0.0
2a-c31	55.4	-68.3	62.0	-30.8	179.8	-178.2	-120.2	-171.5	-35.1	4.1	4.2	0.0	0.0
2a-c32	-66.0	-173.5	87.9	31.4	-174.0	6.3	5.0	-149.8	-32.9	4.1	4.1	0.0	0.0
2a-c33	173.4	164.9	133.2	-71.0	-171.9	5.7	4.4	-153.5	-34.6	4.1	4.3	0.0	0.0
2a-c34	-66.9	-172.9	87.2	32.0	-23.8	-179.6	-120.6	-128.4	-28.9	4.1	4.3	0.0	0.0
2a-c35	40.2	59.8	30.4	-55.4	149.9	176.1	2.8	-162.5	19.3	4.2	4.0	0.0	0.0
2a-c36	55.3	-68.4	61.9	-30.6	179.2	-178.8	119.0	-171.2	-35.1	4.2	4.4	0.0	0.0
2a-c37	162.9	166.9	-55.5	-72.8	-18.4	-179.7	-0.7	-136.9	-33.0	4.4	4.6	0.0	0.0
2a-c38	-53.1	173.3	136.1	-80.4	-21.6	179.9	-0.9	-134.7	-35.1	4.7	5.0	0.0	0.0
2a-c39	-57.6	-76.6	91.5	11.6	-172.2	6.7	4.7	-149.2	-31.6	4.7	4.9	0.0	0.0
2a-c40	71.3	172.9	168.8	-85.0	-22.5	-179.2	-0.3	-117.4	-16.5	5.0	4.6	0.0	0.0
2a-c41	53.9	169.3	105.7	-61.3	169.0	-179.3	-0.2	-137.3	35.2	5.1	5.2	0.0	0.0
2a-c42	-54.7	174.1	145.1	-83.6	-174.3	6.5	2.0	-153.5	-36.2	5.1	5.1	0.0	0.0
2a-c43	64.2	-166.8	62.4	-118.9	-24.3	-178.9	118.8	-124.5	-28.8	5.1	5.4	0.0	0.0
2a-c44	-165.2	-67.2	101.0	-31.4	171.3	-178.9	-1.6	-150.7	-33.3	5.2	5.0	0.0	0.0
2a-c45	64.4	-166.9	62.5	-119.4	-24.6	-179.3	-119.7	-122.6	-28.2	5.4	5.6	0.0	0.0
2a-c46	163.4	166.9	-55.9	-72.7	-17.4	-179.6	119.9	-136.9	-33.0	5.5	5.6	0.0	0.0
2a-c47	61.7	-90.6	64.8	5.1	-16.6	-176.9	-1.1	-108.8	-26.3	5.6	5.6	0.0	0.0
2a-c48	45.0	-168.5	76.4	7.7	-174.7	179.7	0.2	147.0	-38.8	5.7	5.8	0.0	0.0
2a-c49	39.3	59.2	31.5	-54.0	149.4	176.2	-117.8	-163.3	19.0	5.7	5.8	0.0	0.0
2a-c50	162.9	166.9	-55.4	-72.8	-19.1	-179.6	-121.6	-136.0	-32.9	5.7	5.9	0.0	0.0
2a-c51	38.9	58.8	32.0	-53.7	148.1	175.2	122.5	-164.1	18.7	5.7	5.8	0.0	0.0
2a-c52	170.4	177.3	101.3	8.8	173.2	-178.8	-1.5	-155.2	-32.7	5.8	5.8	0.0	0.0
2a-c53	164.2	66.4	-28.6	-74.5	-170.9	5.7	4.8	-150.3	-33.8	5.9	5.8	0.0	0.0
2a-c54	-52.8	173.4	136.4	-80.5	-21.3	180.0	118.7	-134.7	-35.1	6.0	6.4	0.0	0.0
2a-c55	75.5	175.5	171.2	-91.0	-14.0	-179.5	117.9	-132.0	33.0	6.0	6.5	0.0	0.0
2a-c56	-52.7	173.4	135.8	-80.3	-21.5	-179.6	-121.5	-134.1	-35.0	6.1	6.6	0.0	0.0
2a-c57	55.8	-69.0	60.5	-32.9	-171.0	6.2	3.2	-155.6	-35.9	6.2	6.2	0.0	0.0
2a-c58	53.8	169.2	105.7	-61.3	170.5	-178.8	-117.3	-136.9	35.0	6.4	6.6	0.0	0.0
2a-c59	71.2	173.0	168.9	-85.0	-22.3	-178.9	-120.2	-116.4	-16.5	6.4	6.2	0.0	0.0
2a-c60	53.5	169.1	105.2	-61.1	169.2	-179.6	121.5	-136.8	35.1	6.5	6.6	0.0	0.0
2a-c61	-163.8	-65.8	99.3	-32.6	172.8	-177.8	-117.0	-148.3	-32.8	6.7	6.7	0.0	0.0
2a-c62	61.3	96.8	2.9	-71.3	176.5	5.6	1.3	-133.8	31.0	6.7	6.3	0.0	0.0
2a-c63	61.9	-90.7	64.8	5.3	-16.4	-176.7	119.0	-109.2	-26.5	6.8	7.0	0.0	0.0
2a-c64	-164.7	-67.0	100.3	-31.8	171.6	-178.3	121.9	-148.6	-33.1	6.8	7.0	0.0	0.0
2a-c65	154.9	67.1	-36.8	-74.8	-18.8	-179.3	-0.2	-133.8	-31.8	6.9	6.8	0.0	0.0
2a-c66	61.8	-90.7	64.7	4.9	-16.3	-176.5	-121.8	-108.7	-26.3	7.0	7.2	0.0	0.0
2a-c67	44.4	-169.0	76.6	7.8	-174.8	-180.0	-119.1	148.0	-38.8	7.1	7.5	0.0	0.0
2a-c68	44.8	-168.9	76.5	7.6	-176.2	179.2	119.3	148.0	-38.6	7.1	7.5	0.0	0.0
2a-c69	171.0	177.9	100.4	9.2	174.1	-177.8	-116.8	-152.2	-32.1	7.2	7.4	0.0	0.0
2a-c70	170.8	177.6	101.2	8.6	173.1	-178.4	122.2	-152.9	-32.4	7.4	7.7	0.0	0.0
2a-c71	-164.5	-67.0	101.9	-31.2	-175.0	6.4	2.1	-146.2	-35.0	7.8	7.8	0.0	0.0
2a-c72	-152.6	-60.9	75.8	-35.4	-17.8	179.6	-0.2	-129.1	-30.4	8.0	8.3	0.0	0.0
2a-c73	155.0	66.8	-37.0	-74.7	-18.1	-179.3	119.9	-134.4	-31.8	8.1	8.1	0.0	0.0
2a-c74	43.4	-74.0	54.1	-37.6	-17.5	-179.7	-0.4	-137.7	-34.1	8.1	8.1	0.0	0.0

2a-c75	154.0	67.2	-36.9	-74.5	-19.5	-179.3	-121.2	-135.3	-31.9	8.2	8.3	0.0	0.0
2a-c76	170.8	178.2	98.4	10.7	-174.8	6.4	1.6	-148.3	-34.3	8.4	8.7	0.0	0.0
2a-c77	-153.3	-60.8	77.2	-35.3	-16.4	-178.9	118.3	-130.9	-31.3	8.9	9.4	0.0	0.0
2a-c78	-65.3	-171.9	86.0	32.7	-177.1	5.8	148.9	-144.2	-34.8	9.3	9.8	0.0	0.0
2a-c79	177.0	164.7	132.1	-70.6	-174.9	5.6	148.6	-142.9	-35.2	9.4	9.9	0.0	0.0
2a-c80	-151.6	-60.1	74.8	-36.1	-17.9	179.6	-120.0	-128.9	-30.2	9.4	10.0	0.0	0.0
2a-c81	38.4	58.6	32.7	-52.9	154.1	-1.5	-15.9	-168.2	19.1	9.5	9.0	0.0	0.0
2a-c82	42.4	-73.8	54.5	-37.6	-18.1	-179.5	-121.4	-139.0	-34.1	9.5	9.6	0.0	0.0
2a-c83	-56.0	-77.2	90.1	11.4	-176.0	5.8	148.8	-141.8	-33.2	9.9	10.3	0.0	0.0
2a-c84	-53.0	174.5	146.7	-83.9	-172.3	7.2	145.6	-146.9	-37.4	9.9	10.3	0.0	0.0
2a-c85	169.6	64.1	-1.1	-79.8	-170.7	4.6	142.8	-116.9	-28.3	10.5	10.8	0.0	0.0
2a-c86	48.1	-168.2	76.1	7.9	179.0	-3.8	-0.3	142.5	-37.1	10.6	10.5	0.0	0.0
2a-c87	73.4	161.9	111.8	-53.6	-150.1	-174.8	-1.8	88.0	-5.3	10.6	10.3	0.0	0.0
2a-c88	-177.5	61.7	10.8	-105.6	-143.1	-173.1	-3.9	90.0	-10.6	11.0	10.4	0.0	0.0
2a-c89	63.0	-69.1	62.8	-30.2	-169.9	6.0	143.2	-135.6	-35.7	11.2	11.3	0.0	0.0
2a-c90	60.6	95.6	3.9	-69.1	-174.3	8.2	158.0	-134.4	34.2	11.9	12.1	0.0	0.0
2a-c91	81.3	157.4	163.4	-64.1	-153.3	-175.5	-1.4	81.0	-14.1	11.9	11.6	0.0	0.0
2a-c92	73.5	162.1	112.0	-53.9	-150.6	-175.3	117.7	87.4	-5.5	12.1	12.1	0.0	0.0
2a-c93	73.5	161.9	112.0	-53.6	-148.5	-174.4	-122.6	87.1	-5.0	12.2	12.4	0.0	0.0
2a-c94	-164.2	-67.3	102.5	-30.3	-171.6	7.1	143.3	-141.9	-36.5	12.3	12.7	0.0	0.0
2a-c95	-176.7	61.6	11.0	-106.1	-141.9	-172.0	120.1	88.9	-9.4	12.9	12.6	0.0	0.0
2a-c96	-176.6	61.7	10.9	-106.0	-140.3	-171.1	-120.5	89.0	-9.2	12.9	12.6	0.0	0.0
2a-c97	171.9	178.5	97.2	11.5	-171.0	7.4	144.2	-142.3	-35.5	12.9	13.6	0.0	0.0
2a-c98	179.7	62.0	11.8	-100.1	167.5	-2.8	-2.4	78.7	-10.7	13.0	12.9	0.0	0.0
2a-c99	81.5	157.8	163.1	-64.3	-155.4	-176.6	118.2	80.4	-14.4	13.4	13.2	0.0	0.0
2a-c100	81.1	158.0	163.4	-64.4	-152.4	-175.4	-122.4	80.1	-13.7	13.5	13.4	0.0	0.0
2a-c101	83.3	161.2	163.4	-67.4	178.4	-3.0	-4.3	81.1	-16.7	14.3	14.4	0.0	0.0
2a-c102	82.9	160.7	163.3	-66.9	-176.1	-2.6	63.0	81.7	-15.5	16.2	16.2	0.0	0.0
2a-c103	51.3	-168.1	75.8	6.9	168.1	-9.7	-160.7	139.5	-38.8	16.2	16.7	0.0	0.0
2a-c104	179.3	62.1	11.3	-99.9	167.9	-3.4	-138.8	77.9	-11.2	17.1	17.3	0.0	0.0
2a-c105	83.5	162.2	163.8	-68.4	175.6	-4.5	-141.4	80.4	-16.7	18.5	19.0	0.0	0.0

Table S2. Key torsional angles of the considered conformers of (R)-**2b** and the corresponding relative zero-point corrected and Gibbs Free energies, ΔE_{ZPC} and ΔG_{298K}, and resulting Boltzmann populations (popΔE and popΔG). Torsional angles are given in degrees, relative energies in kcal/mol and populations in percentage. The angles are defined in Scheme Sx.

Conf.	α	β	δ	ε	γ	τ	ϕ	ζ	η	ΔE_{ZPC}	ΔG_{298K}	popΔE	popΔG
2b-c1	-60.8	-154.4	87.1	24.6	163.0	179.1	0.9	-146.4	30.9	0.0	0.2	23.7	18.4
2b-c2	177.2	155.9	-51.0	-69.3	166.2	179.3	0.6	-148.1	31.0	0.0	0.0	22.7	27.0
2b-c3	-59.3	-90.7	87.7	15.7	163.9	179.2	1.4	-146.2	30.9	0.3	0.4	13.4	12.5
2b-c4	170.9	81.0	153.6	106.6	166.1	179.1	0.7	-148.0	31.6	0.5	0.6	10.6	9.4
2b-c5	173.6	153.6	133.0	-67.4	179.8	-178.0	-2.1	-162.1	-32.7	1.0	0.8	4.0	6.3
2b-c6	68.1	135.1	-7.8	-75.6	161.1	178.9	0.6	-142.5	32.2	1.1	1.0	3.7	4.6
2b-c7	-66.1	-157.5	86.4	30.2	177.3	-177.6	-2.3	-154.0	-30.0	1.1	1.1	3.6	4.3
2b-c8	167.4	85.5	-31.5	-71.7	179.8	-178.0	-2.3	-162.5	-32.7	1.3	1.2	2.4	3.2
2b-c9	177.1	155.6	-50.9	-69.3	166.6	179.8	-118.3	-147.6	31.1	1.3	1.8	2.3	1.3

2b-c10	176.9	155.6	-50.9	-69.2	166.2	179.0	121.3	-148.1	31.1	1.4	1.4	2.3	2.5
2b-c11	-64.0	-89.4	87.0	21.2	180.0	-177.1	-2.3	-153.4	-27.1	1.6	1.7	1.6	1.4
2b-c12	-61.8	-156.9	87.0	26.6	163.3	-179.7	-116.2	-144.4	30.8	1.6	2.3	1.4	0.5
2b-c13	-61.9	-156.6	86.7	26.9	162.2	179.5	122.8	-144.4	30.9	1.8	2.5	1.1	0.4
2b-c14	170.5	81.3	-33.0	-71.8	166.6	179.4	-118.2	-148.2	31.6	1.8	2.0	1.0	0.9
2b-c15	170.5	82.0	153.8	-71.8	166.4	178.8	121.3	-148.1	31.6	1.8	2.2	1.0	0.7
2b-c16	-58.7	-94.0	87.8	17.0	165.6	-178.6	-113.7	-141.6	30.5	2.1	2.3	0.7	0.5
2b-c17	-59.6	-93.7	87.6	17.9	164.2	-179.6	125.5	-142.3	30.7	2.3	2.8	0.5	0.2
2b-c18	-57.3	163.0	-36.2	89.3	174.3	-178.6	-1.1	-160.5	-34.9	2.3	2.3	0.5	0.5
2b-c19	63.0	125.4	-3.8	-71.6	176.3	-177.7	-2.6	-140.8	-18.2	2.4	1.4	0.4	2.4
2b-c20	173.1	153.7	133.7	-67.8	-179.0	-177.4	-120.1	-161.9	-32.5	2.4	2.4	0.4	0.4
2b-c21	68.8	136.3	-8.4	-76.1	162.0	179.6	-116.3	-141.6	32.2	2.4	2.4	0.4	0.4
2b-c22	173.2	153.7	133.3	-67.6	179.9	-177.9	118.9	-161.3	-32.6	2.4	2.4	0.4	0.5
2b-c23	69.2	136.1	-8.7	-75.9	161.2	178.9	122.9	-141.3	32.4	2.5	2.7	0.3	0.3
2b-c24	-67.4	-158.8	85.9	31.8	178.6	-176.5	-118.0	-151.3	-28.3	2.7	3.1	0.2	0.1
2b-c25	167.1	85.6	-31.6	-71.6	-179.2	-177.6	-120.5	-163.0	-32.7	2.7	2.9	0.2	0.2
2b-c26	167.1	85.1	155.2	-71.6	-179.9	-178.2	118.8	-162.7	-32.6	2.7	2.9	0.2	0.2
2b-c27	-67.4	-158.9	86.1	31.9	176.9	-177.2	120.4	-150.6	-28.6	2.8	3.3	0.2	0.1
2b-c28	54.5	-65.5	63.0	-29.1	159.6	178.1	-0.8	-172.2	-33.0	2.9	3.1	0.2	0.1
2b-c29	-64.5	-91.4	86.4	22.4	179.7	-175.6	-116.3	-147.1	-25.2	3.2	3.6	0.1	0.1
2b-c30	-65.6	-159.1	-90.2	-153.6	-25.0	179.2	0.6	-122.7	-26.8	3.4	3.7	0.1	0.0
2b-c31	-64.9	-91.6	86.4	22.9	179.2	-176.3	122.1	-147.5	-25.4	3.4	4.0	0.1	0.0
2b-c32	63.5	125.9	-4.3	-72.9	177.6	-177.2	-119.2	-140.7	-16.4	3.6	2.6	0.0	0.3
2b-c33	61.9	-155.2	57.2	-119.5	-24.9	-179.1	-0.3	-122.1	-28.5	3.7	3.8	0.0	0.0
2b-c34	67.3	128.6	172.4	-75.2	-22.1	-178.5	-0.6	-114.3	-18.0	3.8	3.5	0.0	0.1
2b-c35	-57.3	166.5	-42.8	91.1	175.5	-176.9	-119.2	-158.5	-34.5	4.0	4.1	0.0	0.0
2b-c36	-57.7	166.7	-42.4	90.5	174.9	-177.7	119.0	-159.2	-34.6	4.1	3.8	0.0	0.0
2b-c37	-64.9	-157.3	86.1	30.2	-173.8	6.4	4.8	-148.8	-32.7	4.1	4.4	0.0	0.0
2b-c38	-169.1	-62.8	100.3	-33.9	171.2	-179.0	-1.3	-152.8	-34.3	4.2	3.6	0.0	0.1
2b-c39	174.2	154.4	132.2	-67.7	-171.9	5.7	4.5	-153.6	-34.4	4.2	4.5	0.0	0.0
2b-c40	53.9	-66.6	62.7	-29.7	162.6	178.1	-116.8	-171.7	-33.6	4.3	4.6	0.0	0.0
2b-c41	-65.0	-157.7	85.4	30.6	-23.5	-179.7	119.4	-125.5	-27.7	4.4	4.9	0.0	0.0
2b-c42	54.3	-65.8	62.2	-29.9	160.8	177.9	121.1	-171.1	-33.3	4.5	4.8	0.0	0.0
2b-c43	-62.3	-90.6	87.0	20.6	-172.3	6.7	4.7	-147.4	-31.1	4.5	4.7	0.0	0.0
2b-c44	167.5	85.9	-32.5	-72.1	-170.6	5.8	4.8	-151.9	-33.9	4.5	4.6	0.0	0.0
2b-c45	164.1	158.9	-55.4	-70.1	-18.5	-179.8	-0.4	-135.2	-32.6	4.7	4.7	0.0	0.0
2b-c46	62.1	-155.3	57.2	-119.2	-24.7	-178.7	119.0	-123.4	-28.9	4.8	5.1	0.0	0.0
2b-c47	-65.1	-159.1	85.3	31.6	-24.8	179.3	-118.4	-121.5	-26.5	5.0	5.8	0.0	0.0
2b-c48	-55.6	160.8	150.2	-84.2	-175.1	6.6	2.4	-153.4	-36.3	5.0	5.3	0.0	0.0
2b-c49	62.2	-155.4	57.3	-119.6	-24.9	-179.0	-120.2	-121.6	-28.4	5.1	5.5	0.0	0.0
2b-c50	66.2	128.7	173.5	-76.9	-22.3	-178.4	-120.9	-115.2	-19.1	5.3	5.2	0.0	0.0
2b-c51	52.9	164.6	105.6	-57.2	169.3	-179.6	0.2	-138.5	35.0	5.3	5.4	0.0	0.0
2b-c52	62.6	120.1	177.8	-70.7	-173.2	6.5	5.1	-138.4	-25.9	5.3	5.0	0.0	0.0
2b-c53	72.2	143.0	171.4	-84.3	-11.6	-179.1	117.7	-132.2	33.2	5.6	5.7	0.0	0.0
2b-c54	-168.4	-63.2	97.9	-33.7	172.7	-178.0	-117.0	-150.6	-34.1	5.6	5.1	0.0	0.0
2b-c55	-51.4	162.4	129.9	-74.9	-22.3	178.4	0.7	-131.0	-34.6	5.7	6.0	0.0	0.0
2b-c56	164.3	159.2	-55.6	-70.1	-17.3	-179.5	119.8	-135.9	-32.7	5.9	6.1	0.0	0.0

2b-c57	163.5	158.8	-55.3	-70.1	-19.1	-179.6	-121.8	-135.6	-32.7	6.1	6.5	0.0	0.0
2b-c58	60.9	-157.1	56.9	66.3	-176.4	5.4	5.5	-134.7	-30.8	6.2	6.5	0.0	0.0
2b-c59	169.9	157.9	-58.3	-67.5	-14.4	179.9	-122.6	-135.5	33.5	6.3	6.9	0.0	0.0
2b-c60	179.6	62.5	21.5	-77.0	-21.1	-179.2	120.1	-113.9	27.0	6.3	6.5	0.0	0.0
2b-c61	-52.7	164.1	138.6	-80.0	-20.7	179.8	118.8	-134.2	-35.4	6.4	6.9	0.0	0.0
2b-c62	-172.6	-90.2	97.7	-18.4	-175.8	6.5	1.5	-147.9	-35.8	6.9	6.9	0.0	0.0
2b-c63	59.2	-112.4	65.9	6.1	-172.9	4.0	3.2	-119.2	-30.0	7.1	7.1	0.0	0.0
2b-c64	-161.4	-48.3	76.2	-38.2	-18.0	179.2	-0.5	-130.5	-33.8	7.1	7.6	0.0	0.0
2b-c65	53.6	-74.5	61.3	-31.9	175.7	4.7	7.1	-158.6	-37.4	7.1	7.6	0.0	0.0
2b-c66	-50.7	163.2	129.9	-75.1	-21.8	178.4	-118.6	-130.1	-34.4	7.2	7.8	0.0	0.0
2b-c67	60.6	-157.8	57.3	65.8	178.8	4.7	-60.6	-134.4	-31.1	8.1	8.5	0.0	0.0
2b-c68	-160.4	-46.8	75.5	-38.7	-17.6	179.4	-119.8	-130.2	-33.4	8.5	8.1	0.0	0.0
2b-c69	-63.3	-155.3	84.6	29.8	-176.5	6.2	147.8	-142.1	-34.3	9.2	9.8	0.0	0.0
2b-c70	178.3	153.4	130.7	-66.5	-174.4	5.7	148.1	-141.8	-34.9	9.4	9.8	0.0	0.0
2b-c71	-60.3	-92.7	85.3	20.4	-175.4	6.3	147.7	-138.4	-32.3	9.5	9.9	0.0	0.0
2b-c72	174.0	80.5	-22.6	-74.6	-171.1	5.3	143.6	-123.7	-30.4	9.8	10.5	0.0	0.0
2b-c73	-54.4	160.8	150.3	-84.0	-172.9	7.3	145.2	-147.0	-37.5	9.9	10.7	0.0	0.0
2b-c74	74.3	150.1	109.0	-41.5	-151.0	-174.4	-2.6	92.1	-3.4	9.9	9.5	0.0	0.0
2b-c75	-173.2	75.6	6.7	-103.4	-145.1	-173.3	-3.8	91.5	-13.7	11.0	10.1	0.0	0.0
2b-c76	58.5	-140.8	66.9	24.0	-172.2	4.7	142.8	-117.2	-29.4	11.6	12.1	0.0	0.0
2b-c77	75.9	146.4	111.0	-41.4	-146.8	-172.1	-118.1	92.5	-1.0	11.9	12.1	0.0	0.0
2b-c78	76.1	147.0	111.1	-41.9	-150.6	-173.3	120.8	92.9	-1.5	11.9	12.2	0.0	0.0
2b-c79	76.4	151.0	110.5	-44.5	176.9	-3.4	-6.2	92.4	-6.2	12.8	12.5	0.0	0.0
2b-c80	51.8	-80.4	62.6	-29.6	174.2	3.5	150.6	-153.9	-39.0	13.0	13.8	0.0	0.0
2b-c81	-171.9	75.4	6.4	-103.6	-145.5	-172.3	120.1	91.0	-12.2	13.0	12.3	0.0	0.0
2b-c82	-171.9	74.3	7.0	-103.4	-140.6	-171.1	-120.4	89.6	-11.2	13.0	12.6	0.0	0.0
2b-c83	-177.0	76.9	2.5	-97.3	167.3	-2.8	-2.7	78.2	-10.7	13.3	13.2	0.0	0.0
2b-c84	75.4	151.2	110.8	-44.9	-175.8	-2.6	61.8	94.2	-4.1	14.6	14.5	0.0	0.0
2b-c85	50.6	-145.0	72.0	-2.4	169.5	-8.3	-167.6	150.6	-38.6	15.0	14.9	0.0	0.0
2b-c86	76.0	97.8	-179.9	-59.8	175.3	-4.9	-143.1	87.2	-18.4	15.3	15.4	0.0	0.0
2b-c87	87.9	148.5	111.5	-43.0	16.6	175.3	0.6	82.1	-2.7	16.0	16.7	0.0	0.0
2b-c88	74.3	-176.5	81.4	5.6	28.3	173.7	-1.6	93.7	-1.3	16.5	16.9	0.0	0.0
2b-c89	90.4	149.7	113.4	-45.6	12.1	174.8	-122.3	81.5	-2.9	17.0	17.5	0.0	0.0
2b-c90	70.7	-172.9	79.8	4.2	38.3	174.8	121.9	96.2	-0.2	17.1	17.5	0.0	0.0
2b-c91	77.1	151.5	112.7	-47.7	173.8	-4.4	-143.3	89.2	-4.4	17.2	17.7	0.0	0.0
2b-c92	-177.1	76.8	2.4	-97.1	167.5	-3.4	-138.4	77.9	-11.2	17.3	17.3	0.0	0.0
2b-c93	86.4	148.7	109.0	-40.1	20.1	174.8	124.8	82.9	-2.7	17.3	18.0	0.0	0.0
2b-c94	77.3	-172.1	79.4	12.4	23.5	173.1	-124.1	89.0	-3.0	17.9	17.7	0.0	0.0
2b-c95	-165.7	70.2	14.6	-109.4	6.5	176.8	4.8	76.3	-10.7	18.7	18.6	0.0	0.0
2b-c96	-173.5	77.9	5.7	-99.7	-7.5	177.9	-117.5	75.5	-5.9	19.2	19.4	0.0	0.0
2b-c97	-163.3	68.4	15.7	-111.1	11.3	175.5	126.3	77.2	-10.9	20.3	20.6	0.0	0.0

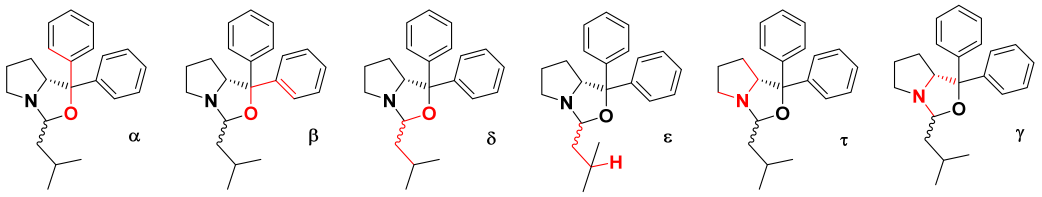

Scheme S2. Angle definition for the oxazolidine (R,?)-**3**.

Table S3. Key torsional angles of the considered conformers of (R,R)-**3** and the corresponding relative zero-point corrected and Gibbs Free energies, ΔE_{ZPC} and ΔG_{298K}, and resulting Boltzmann populations (popΔE and popΔG). Torsional angles are given in degrees, relative energies in kcal/mol and populations in percentage.The torsional angles are defined in Scheme S2.

	α	β	δ	ε	τ	γ	ΔE_{ZPC}	ΔG_{298K}	popΔE	popΔG
3-c1	-165.2	-152.9	175.4	55.6	21.5	21.3	0.0	0.0	66.8	54.6
3-c2	-165.5	-152.6	74.0	43.9	19.4	19.5	0.5	0.2	29.1	39.7
3-c3	-164.6	-151.9	74.8	174.0	20.5	20.6	1.8	1.4	3.0	4.7
3-c4	-165.3	-152.9	159.3	-176.2	22.2	22.3	2.4	2.4	1.1	1.0
3-c5	-165.3	-152.9	-34.9	-36.7	20.3	21.9	4.1	4.2	0.1	0.0
3-c6	-179.9	-142.4	-61.5	43.2	-50.5	45.1	5.5	5.7	0.0	0.0
3-c7	-173.1	-143.9	-51.8	-167.8	-50.7	44.7	9.8	10.0	0.0	0.0

Table S4. Key torsional angles of the considered conformers of (R,S)-**3** and the corresponding relative zero-point corrected and Gibbs Free energies, ΔE_{ZPC} and ΔG_{298K}, and resulting Boltzmann populations (popΔE and popΔG). Torsional angles are given in degrees, relative energies in kcal/mol and populations in percentage.The torsional angles are defined in Scheme S2.

	α	β	δ	ε	τ	γ	ΔE_{ZPC}	ΔG_{298K}	popΔE	popΔG
(R,S)-**3**-c1	-162.8	32.8	-59.2	55.3	22.5	23.1	0.0	0.0	77.9	79.7
(R,S)- **3**-c2	-163.2	-151.2	-159.3	48.9	22.7	23.1	1.0	1.1	13.6	11.4
(R,S)- **3**-c3	-177.5	-148.4	64.1	-31.0	-2.8	-2.3	1.7	1.7	4.4	4.6
(R,S)- **3**-c4	-177.7	-148.2	-73.3	-176.2	-2.7	-1.5	1.9	2.0	2.8	2.4
(R,S)- **3**-c5	-163.3	-151.1	-160.8	177.2	22.8	23.1	2.4	2.2	1.3	1.9
(R,S)- **3**-c6	-178.2	35.1	74.6	-169.6	-2.5	-1.8	4.8	5.1	0.0	0.0
(R,S)- **3**-c7	-173.1	44.5	63.1	39.9	38.2	-44.0	7.6	7.6	0.0	0.0

4. Selected Cartesian coordinates

2a-c1

C	1.74093700	0.74484500	-2.02799600
C	0.40807900	0.96821900	-2.74561800
C	-0.41871100	-0.27255000	-2.36433600
C	0.07059200	-0.67052200	-0.95309300
H	2.45627300	0.19956100	-2.66662500
H	2.22298400	1.68419200	-1.73092300
H	0.53791900	1.06560900	-3.82628100
H	-0.07346200	1.87893800	-2.38169700
H	-1.49399800	-0.09099700	-2.40584400
H	-0.20224000	-1.09405300	-3.05480300
H	0.18241300	-1.75525900	-0.87352800
N	1.40192100	-0.06174200	-0.85869700
C	-0.89621500	-0.27073000	0.23079900
C	-1.01216600	1.25747400	0.31840300
C	-1.99588500	1.97343400	-0.37837100
C	-0.05528700	1.98675200	1.03757500
C	-2.02834700	3.36879100	-0.35229300
H	-2.74914700	1.44509800	-0.95134400
C	-0.08771400	3.38157500	1.07248700
H	0.72021100	1.45391700	1.57361100
C	-1.07514200	4.08076100	0.37666700
H	-2.80271900	3.89593100	-0.90093800
H	0.66282700	3.92054800	1.64262700
H	-1.10233800	5.16553700	0.40311500
C	-2.25907900	-0.98870000	0.12319100
C	-2.40859300	-2.20044100	-0.56949900
C	-3.37753300	-0.50482200	0.82053100
C	-3.62532900	-2.88491200	-0.58371800
H	-1.57961000	-2.63377000	-1.11423000
C	-4.59432800	-1.18763800	0.81572700
H	-3.30463600	0.42057200	1.37952300
C	-4.72701800	-2.38169900	0.10751300
H	-3.70637700	-3.81484400	-1.13784600
H	-5.43654600	-0.78250300	1.36793600
H	-5.67294700	-2.91367800	0.09716900
O	-0.18924400	-0.79594200	1.37839200
C	-0.81064800	-0.72278700	2.65545900
H	-1.65748100	-1.41353100	2.73629900
H	-0.04362800	-1.01335800	3.37747100
H	-1.15321100	0.29141800	2.89222400
C	2.41158400	-0.66157700	-0.13210300
H	2.05346100	-1.40056500	0.57791900
C	3.73360400	-0.39943500	-0.20902900
H	4.10733000	0.35860200	-0.89565000
C	4.76826800	-1.09074500	0.64696400
H	4.24833200	-1.81999000	1.28398900
C	5.49590300	-0.09364100	1.57076600
H	6.01503600	0.67320600	0.98148800
H	4.79007100	0.41626500	2.23507000
H	6.24484200	-0.60123000	2.19097400
C	5.78989200	-1.86100000	-0.21255300
H	6.53915000	-2.36016700	0.41401300
H	5.29546300	-2.62122900	-0.82669200
H	6.32133700	-1.17841600	-0.88814200

2a-c2

C	-0.63084600	-2.61798700	-0.75985100
C	0.75204200	-2.87923500	-1.36336300
C	1.01160700	-1.63102900	-2.22361700
C	0.27706900	-0.47802400	-1.50128100
H	-1.43347700	-3.03650700	-1.38944900
H	-0.74377600	-3.05279200	0.24091700
H	0.77029600	-3.80119700	-1.94989400
H	1.50291700	-2.96858700	-0.57457100
H	2.07148800	-1.42114600	-2.36838000
H	0.56216400	-1.75926500	-3.21385000
H	-0.19265600	0.17321800	-2.24358300
N	-0.74857400	-1.16201000	-0.71275500
C	1.24165900	0.46690900	-0.66433400
C	0.48133500	1.69209100	-0.11112500
C	0.58274100	2.13317600	1.21312800
C	-0.26981400	2.47971200	-1.00097700
C	-0.07328500	3.28983200	1.64561000
H	1.18462800	1.58430300	1.92552600
C	-0.92390200	3.63438800	-0.57683400
H	-0.33276700	2.20391300	-2.04765000
C	-0.83702000	4.04222100	0.75635300
H	0.02357100	3.60089000	2.68116300
H	-1.49686400	4.21815500	-1.29051300
H	-1.34733500	4.93966800	1.09104200
C	1.98156400	-0.35583500	0.39830100
C	1.31231600	-0.85843800	1.52661100
C	3.32478100	-0.71142400	0.22310100
C	1.96723000	-1.67409900	2.44928800
H	0.26766800	-0.62084100	1.68456900
C	3.98507600	-1.52498300	1.14711500
H	3.85890200	-0.36430800	-0.65242500
C	3.31005800	-2.01018000	2.26659000
H	1.42370200	-2.04782700	3.31164100
H	5.02757600	-1.78087200	0.98447300
H	3.82067100	-2.64276700	2.98577700
O	2.15594000	0.92645200	-1.68160400
C	2.98027700	2.04965800	-1.37793300
H	3.75294600	2.07219800	-2.15025900
H	2.41351500	2.98657800	-1.40984600
H	3.45996300	1.95952500	-0.39705100
C	-1.97320600	-0.58565900	-0.44276500
H	-2.00925900	0.48323500	-0.63192100
C	-3.07517100	-1.20414000	0.03210400
H	-3.05845500	-2.27104200	0.24844000
C	-4.37364200	-0.48670200	0.31481000
H	-4.23997500	0.57231700	0.05342400
C	-4.74574200	-0.55953300	1.80910500
H	-3.96610700	-0.10935600	2.43271000
H	-5.68882300	-0.03613900	2.00837400
H	-4.86924400	-1.60260400	2.12757600
C	-5.52447800	-1.04256700	-0.54693100
H	-5.68394700	-2.10861000	-0.34007800
H	-6.46483800	-0.51806000	-0.33803700
H	-5.30389500	-0.93854100	-1.61464500

2a-c3

C	-1.73413200	1.04712800	1.88309300
C	-0.40090500	1.35174300	2.56685600
C	0.41364000	0.06364800	2.35852800
C	-0.07339900	-0.51408100	1.00540500
H	-2.45589100	0.59927100	2.58687500
H	-2.20641900	1.94227900	1.46050400
H	-0.52994000	1.59384900	3.62459700
H	0.09090500	2.20028300	2.08527100
H	1.48881600	0.24495800	2.38315900
H	0.18541400	-0.65640800	3.15121100
H	-0.18446500	-1.60052500	1.08628800
N	-1.40098600	0.08893300	0.83299500
C	0.88820900	-0.25283400	-0.22026300
C	1.08515200	1.25349900	-0.42713900
C	2.15441800	1.94173100	0.16100300
C	0.15388100	1.98971900	-1.17266300
C	2.29042100	3.32325200	0.01345100
H	2.89781500	1.40030900	0.73516500
C	0.29032300	3.37034500	-1.32670500
H	-0.67627000	1.47591000	-1.63892200
C	1.35783300	4.04523200	-0.73205000
H	3.13022500	3.83078600	0.47786600
H	-0.44118800	3.91802800	-1.91322300
H	1.46446100	5.11872700	-0.85256600
C	2.23482200	-0.98935800	-0.09822900
C	2.54051900	-1.90123600	0.91782100
C	3.18501200	-0.79782800	-1.11506000
C	3.76073900	-2.58557800	0.92976900
H	1.83858100	-2.09882700	1.71811100
C	4.39888200	-1.47964200	-1.11089700
H	2.96536600	-0.10721800	-1.92248800
C	4.69548600	-2.37745200	-0.08144200
H	3.97204200	-3.28337100	1.73394200
H	5.11343700	-1.31072800	-1.91048300
H	5.64184300	-2.90869900	-0.07232600
O	0.22277600	-0.70917100	-1.42520700
C	0.16565600	-2.10942500	-1.67151800
H	-0.21445700	-2.67880400	-0.81485000
H	-0.52525800	-2.23619100	-2.50843400
H	1.14464900	-2.51363400	-1.95170500
C	-2.41872100	-0.57098500	0.17561400
H	-2.08501800	-1.40121700	-0.43774000
C	-3.73570500	-0.27517000	0.21027700
H	-4.09704700	0.56539000	0.80027300
C	-4.77956900	-1.03954800	-0.56879600
H	-4.27299400	-1.85304200	-1.10695000
C	-5.82879000	-1.67487200	0.36468000
H	-6.58476000	-2.22733400	-0.20658200
H	-5.36038900	-2.36744000	1.07212900
H	-6.34866700	-0.90275700	0.94630200
C	-5.47257000	-0.14391000	-1.61506500
H	-5.97836400	0.70016900	-1.12883600
H	-4.74740200	0.26556600	-2.32633100
H	-6.22753200	-0.70474600	-2.17958800

(R,R)-3

C	1.67593400	-0.22376900	0.11488900
C	-0.35423000	1.65561800	-2.15644300
H	1.70076900	-1.28991100	-0.15155700
H	-1.08629200	2.19935800	-1.55686700
H	-0.81401900	1.45759300	-3.12795100
N	1.50327100	0.52541400	-1.10986200
C	1.76618900	1.97858500	-1.08223800
H	2.83666800	2.17589500	-1.18306300
H	1.41318200	2.45513100	-0.15443400
C	0.94800500	2.47564500	-2.27292300

H	0.77276700	3.55427900	-2.24145500
H	1.47150600	2.24223200	-3.20615300
C	-0.62517900	-0.06314800	-0.05987800
O	0.46207900	-0.00061200	0.86889600
C	0.07362000	0.34605300	-1.45558900
H	-0.01963200	-0.50598500	-2.12977600
C	2.87277400	0.12935000	0.98307500
H	2.77526300	-0.44368900	1.91403300
H	2.80914100	1.18718900	1.26143700
C	4.25238100	-0.16090300	0.35811000
H	4.30835700	0.36095100	-0.60636000
C	4.48183500	-1.65779300	0.09752400
H	3.75924900	-2.07109500	-0.61309400
H	4.40441000	-2.23237600	1.02951800
H	5.48252500	-1.82685200	-0.31550100
C	5.35904600	0.40321400	1.26138500
H	5.34615100	-0.08319900	2.24524200
H	5.23597200	1.48065200	1.41926500
H	6.34829200	0.23745700	0.82058300
C	-1.71246200	0.90124000	0.43077400
C	-3.01713400	0.84827400	-0.08016500
C	-1.41940600	1.87899300	1.39003600
C	-3.99492800	1.74690000	0.34706200
H	-3.28213200	0.10066000	-0.81889900
C	-2.39893200	2.77441000	1.82581000
H	-0.41883600	1.93434300	1.79966500
C	-3.69183900	2.71554900	1.30568600
H	-4.99606400	1.68416700	-0.06820800
H	-2.14616700	3.51888700	2.57477500
H	-4.45326200	3.41134500	1.64339800
C	-1.17618400	-1.50176100	-0.10287000
C	-1.81481800	-2.02985300	-1.23272700
C	-1.08112200	-2.30816400	1.03926800
C	-2.34131200	-3.32381900	-1.22323600
H	-1.91251000	-1.43814100	-2.13715300
C	-1.59986800	-3.60336200	1.05076000
H	-0.58809700	-1.91645300	1.92138400
C	-2.23416800	-4.11814100	-0.08165700
H	-2.83084800	-3.70843800	-2.11259000
H	-1.50749600	-4.21002100	1.94649400
H	-2.63763400	-5.12568600	-0.07478000

5. Additional references

[1] *Gaussian 09, Rev E.01*, M. J. Frisch, G. W. Trucks, H. B. Schlegel, G. E. Scuseria, M. A. Robb, J. R. Cheeseman, G. Scalmani, V. Barone, B. Mennucci, G. A. Petersson, H. Nakatsuji, M. Caricato, X. Li, H. P. Hratchian, A. F. Izmaylov, J. Bloino, G. Zheng, J. L. Sonnenberg, M. Hada, M. Ehara, K. Toyota, R. Fukuda, J. Hasegawa, M. Ishida, T. Nakajima, Y. Honda, O. Kitao, H. Nakai, T. Vreven, J. J. A. Montgomery, J. E. Peralta, F. Ogliaro, M. Bearpark, J. J. Heyd, E. Brothers, K. N. Kudin, V. N. Staroverov, T. Keith, R. Kobayashi, J. Normand, K. Raghavachari, A. Rendell, J. C. Burant, S. S. Iyengar, J. Tomasi, M. Cossi, N. Rega, J. M. Millam, M. Klene, J. E. Knox, J. B. Cross, V. Bakken, C. Adamo, J. Jaramillo, R. Gomperts, R. E. Stratmann, O. Yazyev, A. J. Austin, R. Cammi, C. Pomelli, J. W. Ochterski, R. L. Martin, K. Morokuma, V. G. Zakrzewski, G. A. Voth, P. Salvador, J. J. Dannenberg, S. Dapprich, A. D. Daniels, O. Farkas, J. B. Foresman, J. V. Ortiz, J. Cioslowski, D. J. Fox, Wallingford CT, USA, **2013**

[2] B. Mennucci, J. Tomasi, R. Cammi, J. R. Cheeseman, M. J. Frisch, F. J. Devlin, S. Gabriel, P. J. Stephens, *J. Phys. Chem. A* **2002**, *106*, 6102-6113.

[3] *CYLview 1.0b*, C. Y. Legault, Université de Sherbrooke, **2009**
